# Supplementary material for: Systematic Review: Drug Repositioning for Congenital Disorders of Glycosylation (CDG)
Source: Int J Mol Sci. 2022 Aug 5;23(15):8725. doi: 10.3390/ijms23158725 (PMC9369176; doi:10.3390/ijms23158725)
Supplement: Supplementary file 1 [file ijms-23-08725-s001.zip › ijms-1813157-supplementary.pdf]

Supplementary file

List of keywords used for literature search on current studies on CDG:

Therapies
Therapy
Therapeutic strategies
Therapeutic options
Acetazolamide
Drug repositioning
Animal models
Yeast
Drosophila melanogaster
Zebrafish
Mouse
Rat
Clinical trials
Induced pluripotent stem cells
Biomarkers
AND
Congenital disorders of glycosylation
ALG1
ALG1-CDG
ALG6
ALG6-CDG
ALG13
ALG13-CDG
ATP6VAP1
ATP6VAP1-CDG
CAD
CAD-CDG
CCDC115
CCDC115-CDG
COG5
COG5-CDG
DOLK
DOLK-CDG
GNE
GNE-CDG
ISPD-CDG
MAGT1
MAGT1-CDG
MPI
MPI-CDG
NANS
NANS-CDG
PGM1
PGM1-CDG
PGM3
PGM3-CDG
PIGM
PIGM-CDG
SL39A8
SL39A8-CDG
SLC35A2

SLC35A2-CDG
SLC35C1
SLC35C1-CDG
SRD5A3
SRD5A3-CDG
TMEM165
TMEM165-CDG
PIGA
PIGA-CDG
PIGO
PIGO-CDG
PMM2-CDG
